# A longitudinal network analysis of pain, psychological processes and psychological and sexual distress in women with endometriosis

**DOI:** 10.1111/bjhp.70071

**Published:** 2026-04-12

**Authors:** Sérgio A. Carvalho, Brittany Patafio, Patrícia M. Pascoal, Teresa Lapa, David Skvarc

**Affiliations:** ^1^ University of Coimbra, Center for Research in Neuropsychology and Cognitive and Behavioral Intervention (CINEICC) Coimbra Portugal; ^2^ Faculty of Health, School of Psychology Deakin University Burwood Victoria Australia; ^3^ Universidade Lusófona, HEI‐Lab: Laboratórios Digitais de Ambientes e Interacções Humanas Lisboa Portugal; ^4^ Clínica Universitária de Psiquiatria e Psicologia Médica, Faculdade de Medicina Universidade de Lisboa Lisboa Portugal; ^5^ PSYLAB, Instituto de Saúde Ambiental (ISAMB), Faculdade de Medicina Universidade de Lisboa Lisboa Portugal; ^6^ Anesthesiology Department Hospitais da Universidade de Coimbra Coimbra Portugal; ^7^ Faculty of Health Sciences Universidade da Beira Interior Covilhã Portugal

**Keywords:** endometriosis, longitudinal, mental health, network analyses, pain, sexual distress

## Abstract

**Objectives:**

Previous studies in endometriosis have linked the experience and impact of pain to psychological distress and sexual dysfunction. However, little is known about how these factors interact over time or how underlying cognitive‐emotional processes contribute to their complex interconnections.

**Design:**

This study followed a Longitudinal Network Approach and explored the interconnections over time between pain (intensity and impact), psychopathological symptoms, sexual distress, and cognitive‐emotional processes (cognitive fusion and difficulties in emotion regulation) in adult women with endometriosis.

**Methods:**

Data was collected in a sample composed of *N* = 210 Portuguese cisgender women with endometriosis in a three‐wave online assessment spanning 12 months.

**Results:**

Using multilevel vector autoregressive network analysis, results showed significant temporal pathways in which pain intensity and pain impact predicted future increases in cognitive fusion, depression, and somatization, indicating a directional cascading effect of physical symptoms on psychological processes. Higher sexual distress over time was associated with decreases in cognitive fusion. Results also found strong positive associations among pain, anxiety, depression, and somatization at each time point, as well as between‐subjects asymmetrical relationships, with anxiety and somatization more strongly predicting sexual distress than the reverse.

**Conclusions:**

Overall, these findings highlight the importance of targeting transdiagnostic cognitive‐emotional processes in interventions to improve pain, mental health, and sexual outcomes in women with endometriosis.

**Statement of Contribution**

**What is already known on the subject?**

- Endometriosis has a multifactorial aetiology, combining genetic, endocrine, inflammatory and immunological mechanisms that contribute to persistent pain and reduced quality of life.
- Endometriosis‐related pain is associated with increased psychological burden, including heightened levels of anxiety, depression, and somatization.
- Difficulties in regulating emotion and maladaptive cognitive processes have been identified as key transdiagnostic processes influencing chronic pain and mental health outcomes.

**What does this study add?**

- The study identifies bidirectional links among pain, anxiety, depression, and somatization, suggesting that these mutually reinforce one another rather than act independently.
- Endometriosis pain prospectively predicts increases in cognitive fusion, depression, and somatization, revealing a cascading influence of physical symptoms on psychological functioning.
- The dynamic interconnections among emotion regulation, cognitive fusion, pain, and psychological and sexual distress highlight that cognitive‐emotional transdiagnostic processes may serve as intervention targets to alleviate pain and distress in endometriosis.

## INTRODUCTION

Endometriosis is a chronic gynaecological neuro‐inflammatory condition in which tissue similar to the lining of the uterus grows ectopically (Capezzuoli et al., 2020). Endometriosis has a multidetermined etiopathogenesis (e.g., genetic, endocrine, inflammatory, immunologic). It affects approximately 10% of women of reproductive age (Zondervan et al., 2020), with a significant detrimental impact on mental health and functioning (e.g., Laganà et al., 2017).

### Pain, psychological and sexual distress in endometriosis

Pain is a core symptom of endometriosis, influenced by stress, hormonal disruptions, and inflammation (Appleyard et al., 2020; Markham et al., 2019). Common pain experiences include dysmenorrhea, chronic pelvic pain (approximately 60% of women with endometriosis report chronic pelvic pain; Ballard et al., 2008), and discomfort during intercourse, with significant detrimental effects on women's daily lives (Giudice & Kao, 2004; Simoens et al., 2012). The impact of endometriosis on women's sexual health (e.g., sexual symptoms) has been recently examined and seems to be influenced by biological (e.g., the functional impact of surgical procedures) and psychological factors (e.g., cognitive‐emotional processes, such as emotion regulation skills) (Vannuccini et al., 2023), including, but not limited to, endometriosis‐related pain. Indeed, the experience of sexual distress (i.e., experiencing negative emotional response associated with sexual function and/or sexual activity as a whole; Stephenson & Meston, 2010) can result from the interaction of multiple factors (e.g., biological, socioeconomic, religious, political, cultural; e.g., Raposo et al., 2023, 2024) and, although dyspareunia is one contributing factor (e.g., Del Forno et al., 2024), so are endometriosis‐related mental health symptoms (e.g., depression, anxiety), body dissatisfaction and negative self‐image (e.g., Fritzer et al., 2013; Van Niekerk et al., 2022). Overall, while a few studies have suggested the relationship between pain, mental health and sexual distress and/or dysfunction in endometriosis, the extent of this relationship and how these multiple factors mutually relate, require further examination, not only because dyspareunia does not seem to be a determining factor of sexual distress in endometriosis (Barbara et al., 2017) but also because pain itself is a multifactorial symptom (e.g., Linton et al., 2018). In fact, research suggests that the pain (e.g., pain intensity) experienced by those with endometriosis is associated with psychological processes involved in the experience and regulation of emotions (see Kalfas et al., 2022), which calls for a transdiagnostic and process‐based approach to the psychological and sexual distress in endometriosis.

### Transdiagnostic and process‐based approaches as a useful framework

Transdiagnostic and process‐based approaches are useful theoretical frameworks to explore the biopsychosocial factors contributing to the mental health of chronically ill patients by emphasizing shared mechanisms and psychological processes rather than discrete diagnoses or outcomes. The transdiagnostic approach (Dalgleish et al., 2020) identifies common biopsychosocial processes—such as emotion (dys)regulation—that underlie diverse disorders and symptomatology. It provides a flexible, mechanism‐focused understanding of comorbid and heterogeneous presentations common in chronic illness, such as endometriosis (Payne & Barreveld, 2023) and chronic pain (Linton, 2013). The process‐based approach is a dynamic idiographic framework that highlights individualized, functionally oriented conceptualization of mental health, focusing on psychological processes rather than on symptomatology (Hayes et al., 2020). It emphasizes processes such as emotional flexibility, cognitive reappraisal and values‐based action in mental health and reconceptualizes health‐related quality of life (HRQL) as a dynamic, goal‐directed adaptation process (Nolan & Sharpe, 2023). Together, transdiagnostic and process‐based approaches are complementary and converge in focusing on adaptive, individualized, and mechanism‐focused conceptualization and care that aligns treatment with the lived experience of chronic illness.

### Emotion regulation: A Core transdiagnostic psychological process in endometriosis

Emotion regulation (ER) is a transdiagnostic psychological process that, although lacking a scientific consensus on its definition (see Tull & Aldao, 2015), is commonly conceptualized as the ability to manage emotional experiences and expressions to respond effectively to environmental demands (see Gross, 1998). It plays a crucial role in both mental and physical health, particularly in the context of chronic illnesses (Wierenga et al., 2017). Research indicates that ER affects mental health, pain perception, and the persistence of pain, making it particularly relevant in conditions like endometriosis (e.g., Koechlin et al., 2018; Ruiz‐Aranda et al., 2010). Strategies for regulating emotions may help predict the quality of life in individuals with endometriosis (e.g., Márki et al., 2017; Rodríguez‐Lozano et al., 2022). The relationship between different ER skills, pain and mental health has been examined in endometriosis, suggesting that adaptive ER skills (e.g., cognitive reappraisal, acceptance, problem‐solving) are associated with good mental health parameters (e.g., Bernini et al., 2022; Zarbo, Brugnera, Compare, et al., 2019; Zarbo, Brugnera, Secomandi, et al., 2019), while maladaptive ER skills (e.g., catastrophizing, avoidance, rumination) are associated with poor mental health (e.g., Facchin et al., 2017; Levang & Pukall, 2024).

### Beyond emotion regulation: The role of cognitive fusion as a core process of distress

Although difficulties in regulating emotions seem to contribute to pain and mental health outputs in endometriosis (e.g., Gevaudan et al., 2023), studies have not examined the cognitive processes associated with difficulties in ER. Cognitive fusion (i.e., the tendency to get entangled with one's internal experiences instead of looking at them as transient internal events; Hayes et al., 2006) is a transdiagnostic core process of inflexible responding and central to process‐based psychological approaches (Hayes & Hofmann, 2017), seemingly cutting across different cognitive processes (e.g., catastrophizing, rumination, hopelessness) relevant to pain‐related clinical conditions (McCracken & Morley, 2014). Although empirical literature suggests that cognitive fusion is a crucial psychological process for understanding the trajectory of mental health in chronic pain conditions (e.g., Carvalho et al., 2019), its contribution to ER difficulties in endometriosis is, to our knowledge, unexamined.

### Towards dynamic models of psychological phenomena in endometriosis: The use of network analysis

The bidirectional and self‐perpetuating nature of psychological phenomena requires innovative statistical approaches to examine complex inter‐relationships between variables over time. The network approach in psychology posits that symptoms are interconnected and influence one another, contributing to the emergence of mental and physical health challenges (Borsboom et al., 2021). Unlike traditional methods that analyse variables in isolation, network analysis enables the examination of dynamic relationships through partial correlations, visually depicting connections among variables. This methodology is particularly useful for uncovering the relationships between intercorrelated items and identifying potential causal pathways when used with longitudinal data. It has gained traction in clinical and health psychology for studying comorbidity, allowing for a more nuanced understanding of psychological constructs (Borsboom & Cramer, 2013).

The current study uses a network approach to analyse the longitudinal interconnectedness of pain (intensity and impact), psychological distress (depression, anxiety, somatization), sexual distress, and cognitive‐emotional processes (cognitive fusion and difficulties in ER) in women with endometriosis.

## METHOD

### Participants

The current sample is composed of 210 (cisgender) women, aged 22–53 (*M* = 36.27 years, *SD* = 7.28), with a prior diagnosis of endometriosis. The diagnosis was self‐reported, and all participants reported having had their diagnosis of endometriosis conducted by a medical doctor of gynaecology. Participants completed an online battery of sociodemographic, medical, and psychological questionnaires at three time points over 12 months. We have chosen three measurements across 12‐months because these timings reflect a naturalistic observational approach, that is, the constructs being measured are largely considered to be stable, and the use of other methods of measurement (e.g., Ecological Momentary Assessments; EMA) would not yield meaningful variations over the timeframes most common in EMA (i.e., multiple daily measurements over a one or two week period).

Table 1 presents our sample characteristics. The sample was female, with an average mid‐thirties age, and the majority in a marriage or civil union. The sample was generally highly educated, with over two‐thirds completing tertiary education, and currently employed, with *n* = 10 on sick leave (*n* = 6 due to endometriosis, and *n* = 4 due to pain unrelated to endometriosis).

**TABLE 1:** Participants' sociodemographic and medical characteristics (*N* = 210).

	M	SD	Min	Max			
Age (years)	36.27	7.28	22	53			
Unemployed (months)	39.13	55.58	1	180			
Sick leave (months)	8.8	9.7	1	33			
Endometriosis onset (years)	20.28	8.78	9	50			
First consultation (years)	23.35	8.33	10	50			
Diagnosis age (years)	31.41	6.1	19	50			
		***N***	**%**			***N***	**%**
Marital status	Single	70	33.3%	Endometriosis Sick leave	No	4	40%
	Married or Civil Union	130	61.9%		Yes	6	60%
	Separated	1	0.5%	Pain sick leave	No	6	60%
	Divorced	9	4.3%		Yes	4	40%
	Widowed	0	0%	Reproductive	No	120	57.1%
				Plan	Yes	90	42.9%
Employment	Working	176	83.8%	Income	0–5 k	25	11.9%
	Unemployed	17	8.1%		5–10 k	25	11.9%
	Sick leave	10	4.8%		10–13.5 k	29	13.8%
	Retired	3	1.4%		13.5−17.5 k	27	12.9%
	Student	4	1.9%		19–23.5 k	9	4.3%
Education	Middle school	4	1.9%		23.5−27.5 k	33	15.7%
	Secondary School	7	3.3%		27.5 L–32.5 k	27	12.9%
	High school	51	24.3%		32.5–40 k	14	6.7%
	Bachelors	63	30%		40–50 k	10	4.8%
	Post‐Grad	29	13.8%		50–100 k	11	5.2%
	Masters	53	25.2%				
	Doctorate	3	1.4%				
		*N*	%			*N*	%
Pain symptoms				Pain periodicity			
Dysmenorrhea	No	33	15.7%	No pain		5	2.4%
	Yes	177	84.3%	Monthly pain		68	32.4%
Pelvic pain	No	54	25.7%	Weekly pain		41	19.5%
	Yes	156	74.3%	Daily pain		54	25.7%
Middle pain	No	102	48.6%	Constant pain		42	20.0%
	Yes	108	51.4%				
Dyspareunia	No	51	24.3%	Endometriosis treatments			
	Yes	159	75.7%	Hysterectomy		19	9.0%
Other pain	No	125	60.0%	Analgesia medication		178	84.8%
	Yes	84	40.0%	Hormone therapy		163	77.6%
				Physiotherapy		47	22.4%
Other symptomatology				Surgeries		126	60.0%
Fatigue		167	79.5%	Other		11	5.2%
Abdominal bloating		188	89.5%				
Chronic pain (unrelated to endometriosis)		36	17.1%				
Sexual problems		63	30.0%				

### Procedures

Participants were invited to participate in the current three‐wave study via social media websites and national associations for individuals with endometriosis. Interested participants were directed to a secure online survey (Limesurvey) and completed a battery of online questionnaires. Before completing the survey, participants provided informed consent and were made aware of the purpose of the study and the confidentiality of their data. Inclusion criteria were: (a) assigned female at birth; (b) >18 years of age; (c) previous diagnosis of endometriosis conducted by an obstetrics‐gynaecology medical doctor; (d) Portuguese nationality. The exclusion criterion was being currently pregnant. Participants were invited to respond to the same battery of online questionnaires in three assessment moments: baseline (T0), 6 months later (T1), and 12 months later (T2). The survey was accessed by 248 individuals, of whom 230 completed the battery of questionnaires at T0. Twenty participants were excluded for not meeting eligibility criteria: *n* = 10 participants did not report their endometriosis being diagnosed by an obstetrics‐gynaecology medical doctor, *n* = 3 participants were pregnant, *n* = 1 participant was duplicate (we excluded the responses from the second time), and *n* = 6 participants assigned female at birth were transgender or non‐binary. Data collection occurred from April 2023 to May 2024. The final databases comprised *n* = 210 at T0, *n* = 122 at T1 and *n* = 112 at T2.

This study was approved by the University's Scientific and Ethics Committee, where the first author is conducting his research, on the 22nd of February 2023 (Ref number: CEDI/FPCEUC:72/7).

### Instruments

#### Pain intensity

The Numerical Pain Rating Scale (NRS; Hartrick et al., 2003; Ferreira‐Valente et al., 2011) is an 11‐point scale used to measure pain intensity, ranging from 0 (“No pain”) to 10 (“Worst imaginable pain”). It assesses pain through three ratings: current pain, highest pain in the last 24 h and lowest pain in the last 24 h, which are then averaged to provide a single score of pain intensity. The reliability of this scale in the current study was good: Cronbach's alpha (α) = .88.

#### Endometriosis pain impact

The Endometriosis Health Profile (EHP‐30; Jones et al., 2001; Portuguese validation: Nogueira‐Silva et al., 2015) is the most used self‐report measure of health‐related quality of life in endometriosis, composed of 30 items that assess different dimensions of the impact of endometriosis on quality of life. Items are ranked using a 5‐point Likert scale from 0 (“Never”) to 4 (“Always”), where higher scores mean a worse endometriosis‐related quality of life. For this study, we used the dimension “Pain impact” which assesses the impact of endometriosis‐related impact on quality of life (e.g., “Been unable to do jobs around the home because of the pain?”). Internal consistency was excellent in this study: α = .96.

#### Psychopathological symptoms

The Brief Symptom Inventory (BSI; Derogatis, 2001; Portuguese version: Canavarro, 2007) is a 53‐item measure of different dimensions of psychological distress (9 symptom dimensions and 3 global indices). Items are ranked using a 5‐point Likert scale ranging from 0 (“Never”) to 4 (“Very often”), where higher scores mean greater psychological distress. In this study, we used the Portuguese 19‐item version that measures the three most common dimensions of psychological symptoms: depression (e.g., “feelings of worthlessness”), anxiety (e.g., “Nervousness or shakiness inside”) and somatization (e.g., “Pains in the heart or chest”). Internal consistency was good for depression (α = .89), anxiety (α = .84), and somatization (α = .82).

#### Sexual distress

The Sexual Distress Scale – Revised (SDS‐R; DeRogatis et al., 2008; Portuguese validation: Tavares et al., 2022) is the revised version of the female sexual distress scale with an additional item that assesses low sexual desire (“Are you bothered by low sexual desire?”). The measure consists of 13 items that evaluate sexual distress on a one‐dimensional scale using a 5‐point Likert scale, where responses range from 0 (“Never”) to 4 (“Always”). Higher scores indicate greater levels of sexual distress. This study found an excellent internal consistency: α = .96.

#### Cognitive fusion

The Cognitive Fusion Questionnaire (CFQ‐7; Gillanders et al., 2014; Costa et al., 2017) is a 7‐item tool that measures cognitive fusion, with items rated from 1 (“Never true”) to 7 (“Always true”). An example item is “I get so caught up in my thoughts that I am unable to do the things that I most want to do”. The current study found excellent internal consistency (α = .95).

#### Emotion (dys)regulation

The Difficulties in Emotion Regulation Scale – Short Form (DERS – SF; Kaufman et al., 2016; Portuguese version: Moreira et al., 2022) is an 18‐items version of the DERS scale that assesses difficulties in regulating emotions in 6 different dimensions, 3‐items per dimension, measured in a 5‐point Likert scale rated from 1 (“Almost never”) to 5 (“Almost always”). The dimensions examined include (1) Nonacceptance of Emotional Responses (e.g., “When I'm upset, I feel guilty for feeling that way”), (2) Difficulties Engaging in Goal‐Directed Behaviour (e.g., “When I'm upset, I have difficulty concentrating”), (3) Impulse Control Difficulties (e.g., “When I'm upset, I become out of control”), (4) Lack of Emotional Awareness (e.g., “I pay attention to how I feel”), (5) Limited Access to Emotion Regulation Strategies (e.g., “When I'm upset, it takes me a long time to feel better”) and (6) Lack of Emotional Clarity (e.g., “I am confused about how I feel”). Although Gross's model and definition of ER are the most commonly used, its focus on the different points of emotion‐generative processes (i.e., antecedent‐ versus response‐focused) is not the best approach to follow in this study, which was more focused on the positive (adaptive) and negative (maladaptive) ER strategies and abilities (Tull & Aldao, 2015). This scale can be used unidimensionally to measure difficulties in emotion regulation, where higher scores mean more difficulties in emotion regulation. The Portuguese validation study found that the total score should be computed excluding the 3 items composing the “lack of emotional awareness” factor; thus, we followed that instruction in this study. We found good internal consistency for this scale (α = .92).

### Data analyses

#### Descriptive statistics

Sociodemographic and clinically relevant information was examined with descriptive analyses, and the internal consistencies of the instruments were analysed according to Cronbach's alpha, where α > .70 were considered acceptable (Field, 2013). These analyses were conducted in the SPSS software (v.29.0.2.0; IBM Corp, 2023).

#### Network analysis

Network analysis is a statistical method for data‐driven exploration of relationships among psychological constructs and symptoms (Epskamp et al., 2018), focusing on the organization of components within a system and the relationships present within such (Borsboom et al., 2021). Network analysis can assist researchers in identifying otherwise hidden relationship patterns within complex systems, as well as uncovering the most influential variables (“nodes”) within a network (Epskamp et al., 2012). Network analyses can be utilized for both cross‐sectional and longitudinal (referred to as “panel”) data, with the latter being relevant to the current study. Network analyses for longitudinal data allow for both the association structure of variables at a given time point and the way these conditional dependencies' change over time to be analysed, thus allowing for consideration of within‐person (i.e., temporal effects and contemporaneous effects) and between‐person (i.e., between‐subjects effects) variance within the network structure. Effects over time are examined through temporal effects, whereby relationships within the temporal network indicate that a node (i.e., a variable) predicts another node at the next measurement. For cases where there are more than two measurements, the prediction between nodes is made from the first measurement to the average of the remaining measurements.

### Analysis

Analyses were performed using the mlVAR package (Epskamp et al., 2024) in R (R Core Team, 2024). We specified our network model as having orthogonal temporal effects and correlated contemporaneous effects and used random effects for both. Lag was set to one, and estimates were standardized and scaled within‐person. Visual and statistical inspection revealed that missing data were missing completely at random (Littles MCAR (72) = 89.5, *p* = .79), and so multiple imputation was used to replace missing values where participants had not dropped out of the sample (Woods et al., 2024). Temporal effects represent the standardized regression coefficient from a variable at earlier measurements to future, representing the average effect for that variable across the second and third measurements. Contemporaneous effects are defined as within‐person associations averaged over all measurements. Between‐subjects effects are defined as the associations at any given time across participants.

### Sample size and power analysis

Recommendations for sample size in autoregressive models suggest that large numbers of repeated measurements are desirable, though this can be compensated for in models with larger samples and intraclass correlations approaching zero (Hecht & Zitzmann, 2021b). Our three measurements, with a sample size of 210 and intraclass correlations ranging from .36 to .45 across outcomes, fall generally within the poor to fair range and are subject to bias of around 10%. As such, estimates with a significance greater than .01 should be treated with caution.

## RESULTS

Our sample was entirely female, aged in the mid‐thirties, and in a marriage or civil union. This sample presents an average of approximately ten years from the onset of endometriosis symptoms (*M* = 20.28; *SD* = 8.78) to the formal diagnosis (*M* = 31.41; *SD* = 6.10). More than half of the participants reported not having accomplished their reproductive plan (i.e., wanting to have [more] children). Most participants reported having endometriosis‐related pain daily or monthly, namely dysmenorrhea, pelvic pain, middle pain, and dyspareunia. Other symptoms and/or clinical problems reported by most participants included fatigue and abdominal bloating, with some participants reporting other chronic pain diagnoses and sexual problems. Most participants reported using analgesia medication and hormone therapy for endometriosis management, as well as having had surgeries. Table 2 presents the descriptive statistics for our outcome variables over time.

**TABLE 2:** Descriptive statistics for outcome variables over time.

	T0				T1				T2			
	M	SD	Min	Max	M	SD	Min	Max	M	SD	Min	Max
Anxiety	9.16	5.44	0	23	8.20	5.12	0	20	8.10	5.63	0	21
Depression	9.45	5.48	0	24	8.83	6.01	0	22	8.53	6.04	0	23
Cognitive fusion	28.92	9.82	7	49	28.63	11.08	7	49	28.38	9.29	7	49
Emotion regulation	2.35	0.83	1	4.71	2.29	0.80	1.07	4.64	2.34	0.80	1	4.79
Endometriosis pain	43.84	23.42	0	95.45	34.76	23.50	0	93.18	34.09	21.64	0	84.09
Pain intensity	3.15	2.45	0	8.67	2.84	2.59	0	9	2.72	2.41	0	8.67
Sexual distress	26.40	12.70	0	52	27.70	13.05	0	52	27.03	14.26	0	52
Somatization	9.35	5.69	0	25	8.05	5.44	0	21	8.15	6.35	0	26

*Note*: T0 = baseline, T1 = 6 months after baseline, and T2 = 12 months after baseline.

### Effects across time (temporal effects)

**FIGURE B1:**
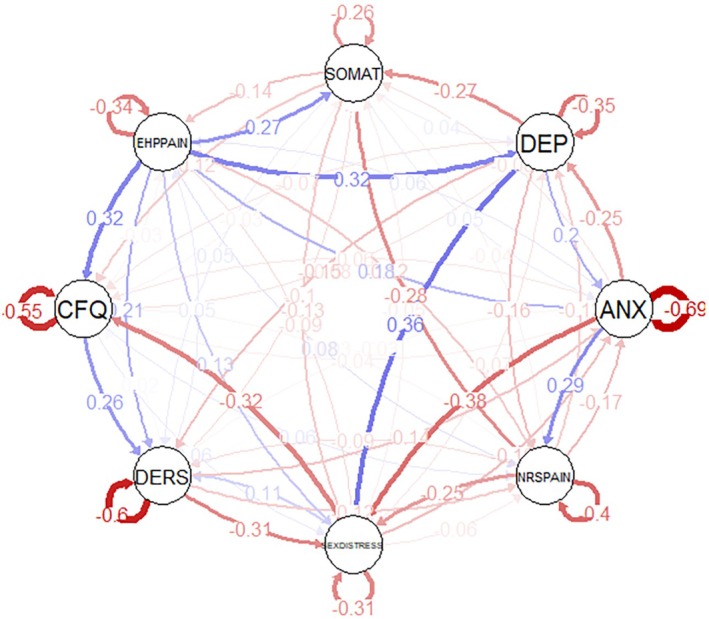
Temporal network visualization. Positive edges are solid red lines; negative are blue. Anx, Anxiety; CFQ, Cognitive Fusion; Dep, Depression; DERS, Difficulties in Emotion Regulation; EHPPain, Endometriosis Pain Impact; NRSPain, General Pain Intensity; Sexdistress, Sexual Distress; Somat, Somatization.

Table 3 shows the significant temporal effects in the network analysis, with the complete table (inclusive of non‐significant effects) shown in Appendix A. The visualization of the temporal network is shown in Appendix B (Figure B1). As shown in Table 3, all autoregressive pathways (B) except for depression are significant and negative, indicating that most of the outcomes tended to decrease over the three measurements. We observe seven cross‐lagged effects, wherein sexual distress is negatively associated with cognitive fusion over time, endometriosis pain is positively associated with cognitive fusion, depression and somatization over time, difficulties in emotion regulation and pain intensity are negatively associated with sexual distress, respectively, over time and somatization is negatively associated with pain intensity over time. There was also evidence of significant inter‐individual variability, as reflected in the presence of random‐effects standard deviations for all associations. When examining the significant cross‐lagged temporal effects, there were potential indirect (mediation) effects present in the data that may warrant further examination. These potential mediation effects are outlined in Appendix C, Tables C1, C2, C3.

**TABLE 3:** Significant temporal effects in the network analysis.

Predictor variable (from)	Temporal dependent variable (to)	B	SE	*p*	*t*	Random effects SD
Autoregressive pathways						
Anxiety	Anxiety	−.688	.190	< .001	−3.62	.219
Emotion regulation^a^	Emotion regulation^a^	−.596	.146	< .001	−4.08	.232
Cognitive fusion	Cognitive fusion	−.548	.106	< .001	−5.17	.177
Pain intensity	Pain intensity	−.401	.127	.002	−3.16	.256
Sexual distress	Sexual distress	−.314	.123	.011	−2.55	.226
Endometriosis pain	Endometriosis pain	−.337	.143	.019	−2.36	.261
Somatization	Somatization	−.263	.131	.045	−2.01	.242
Cross‐lagged effects						
Sexual distress	Cognitive fusion	−.318	.096	.001	−3.31	.184
Endometriosis pain	Cognitive fusion	.320	.103	.002	3.11	.170
Endometriosis pain	Depression	.321	.139	.021	2.31	.262
Emotion regulation^a^	Sexual distress	−.308	.135	.023	−2.28	.219
Somatization	Pain intensity	−.283	.134	.035	−2.11	.259
Pain intensity	Sexual distress	−.253	.124	.040	−2.04	.220
Endometriosis Pain	Somatization	.269	.135	.046	1.99	.243

*Note*: B represents the standardized regression coefficient *from* a variable at earlier measurements *to* future, representing the average effect for that variable across the second and third measurement.

^a^ Difficulties in emotion regulation.

### Effects at any time (contemporaneous and between‐subjects effects)

Table 4 provides information on the directionality of each comparison, as well as their partial (considering other relationships in the network) and raw (bivariate) correlations.

**TABLE 4:** Contemporaneous and between‐subjects effects in the network analysis.

Variable 1	Variable 2	Effect	Directionality		Partial *r*	*r*
			1‐>2	1<‐2		
Endometriosis pain	Somatization	C	.656	.710	−.045	.052
		B	.156	.590	.162	.937
	Pain intensity	C	.988	.630	.021	−.002
		B	0	0	.690	.958
	Cognitive fusion	C	.777	.689	−.036	−.030
		B	.209	.572	.146	.887
	Emotion regulation	C	.398	.186	.122	.111
		B	.437	.288	−.156	.890
	Depression	C	.690	.623	.048	.127
		B	.831	.520	.046	.902
	Sexual distress	C	.076	.142	−.183	−.130
		B	.962	.507	.059	.912
	Anxiety	C	.013	.013	.267	.290
		B	.690	.852	−.01	.909
Cognitive fusion	Depression	C	.854	.986	.008	.164
		B	.082	.003	.414	.988
	Emotion regulation	C	.191	.191	.138	.213
		B	.134	.007	.342	.989
	Anxiety	C	.649	.353	.076	.122
		B	.506	.859	.082	.985
	Sexual distress	C	.038	.034	.242	.316
		B	.444	.894	.078	.974
	Somatization	C	.970	.810	−.014	.114
		B	.861	.306	.110	.971
	Pain intensity	C	.359	.267	.126	.129
		B	.116	.047	−.329	.840
Sexual distress	Pain intensity	C	.923	.645	.037	.059
		B	.683	.177	.156	.884
	Anxiety	C	.965	.913	−.010	.121
		B	.565	.978	.056	.981
	Depression	C	.018	.017	.243	.336
		B	.360	.980	.083	.976
	Somatization	C	.666	.265	.093	.228
		B	.599	.890	.038	.973
Emotion regulation	Somatization	C	.036	.047	.216	.309
		B	.547	.162	−.193	.973
	Anxiety	C	.599	.600	−.060	.146
		B	.002	.003	.578	.992
	Depression	C	.122	.141	.164	.304
		B	.263	.531	.16	.986
	Sexual distress	C	.226	.185	.135	.264
		B	.401	.021	.283	.980
	Pain intensity	C	.555	.670	−.058	−.029
		B	.316	.916	.076	.856
Pain intensity	Depression	C	.992	.981	−.002	.003
		B	.776	.679	−.068	.863
	Anxiety	C	.882	.798	−.023	−.011
		B	.954	.735	.032	.878
	Somatization	C	.753	.784	−.006	−.010
		B	.092	.261	.273	.911
Anxiety	Somatization	C	.039	.052	.211	.310
		B	.088	.002	.427	.984
	Depression	C	.002	.003	.322	.417
		B	.733	.964	.034	.986
Depression	Somatization	C	.228	.217	.136	.328
		B	.175	.077	.291	.979

*Note*: C = Contemporaneous effects, B = Between‐subjects effects.

#### Contemporaneous effects

We observe that many variables have bivariate directional contemporaneous effects, such as pain intensity with somatization, depression, and anxiety; sexual distress with anxiety and pain intensity; and cognitive fusion with depression and somatization. We observed some evidence of partial correlations between variables ranging from trivially small to more than the recommended minimally practical effect (RMPE) size (Ferguson, 2016). However, other variables in the network showed a lack of temporal (direct) effect (e.g., depression and anxiety with somatization), yet robust raw and partial correlations.

#### Between‐subjects effects

When considering the between‐subjects effects, we observe general stability across the variables when examining raw correlations. This either indicates multicollinearity between the variables or, more likely, that participants are experiencing relationships in very similar ways. We observe some indication of strong reciprocal between‐subjects direct effects, for example, anxiety and depression, and anxiety and pain intensity, and unbalanced directional effects; for example, somatization and anxiety are strongly related to sexual distress, but sexual distress is only moderately associated with somatization and anxiety. This is also true for sexual distress and cognitive fusion, and endometriosis pain and sexual distress. Similarly, depression is strongly related to sexual distress, however sexual distress is only weakly associated with depression; and the same is true for pain intensity and emotion regulation, and cognitive fusion and somatization.

## DISCUSSION

This study followed a longitudinal network approach aiming to explore the dynamic interconnectedness among pain, psychological distress, sexual distress, and cognitive‐emotional processes in women with endometriosis over a 12‐month period. Our findings show several key pathways and reciprocal associations, providing new insights into the complex experience of women living with endometriosis.

The temporal network (Table 3) showed robust cross‐lagged effects, highlighting how endometriosis‐related pain positively predicted future levels of cognitive processes and psychopathological symptoms, such as cognitive fusion, depression, and somatization. This suggests that the intensity of pain experienced by endometriosis patients, as well as the impact on the quality of life of endometriosis pain, potentially contributes to a cascading impact on future psychological functioning. Further, while temporal effects suggested pain intensity was a key driver of subsequent outcomes, we observe that contemporaneous measurements of depression, somatization, and cognitive fusion share strong and bidirectional associations with endometriosis pain, indicating that changes over time tend to occur in clusters of intercorrelated effects. For example, pain intensity and depression showed strong reciprocal associations both within time points and across individuals, suggesting a cyclical association rather than a unidirectional effect. Similarly, ER and anxiety, as well as anxiety and somatization, demonstrated patterns consistent with mutual reinforcement. These findings suggest that psychological and somatic processes in endometriosis are not strictly causal in one direction, but rather may evolve in tandem, amplifying each other over time. These results are aligned with existing studies that show that chronic pain conditions, including endometriosis, are intertwined with maladaptive cognitive patterns and emotional difficulties (Eriksen et al., 2008; McCracken & Morley, 2014) and that ER strategies and abilities are especially involved in the experience of pain, psychological distress and quality of life in women with endometriosis (Carvalho et al., 2025). Although prior research show that cognitive processes are involved in endometriosis pain (e.g., Kalfas et al., 2022), to our knowledge this is the first study to explore the interconnectedness and temporal bidirectionality of pain and cognitive fusion (which is conceptualized as an underlying transdiagnostic process of a range of cognitive phenomena; Hayes et al., 2006), and psychopathological symptoms in endometriosis. In particular, the longitudinal relationship between endometriosis pain and cognitive fusion corroborates the hypothesis that when women become entangled with their internal experiences (e.g., somatic symptoms; physical sensations), it may contribute to rigid cognitive and emotional responses, limiting their ability to flexibly engage with life and exacerbating distress over time (Hayes et al., 2006; Hayes & Hofmann, 2017).

Additionally, and somewhat counterintuitively, sexual distress showed a unique negative longitudinal association with cognitive fusion. This suggests that, over time, higher sexual distress was linked to reduced cognitive fusion. One possible interpretation of this unexpected finding can be drawn from the theoretical underpinnings of cognitive fusion (i.e., Acceptance and Commitment Therapy; Hayes et al., 2006): this negative relationship may reflect a form of disengagement or distancing from entangled cognitive patterns, possibly as a protective adaptation, that is, it may be speculated that experiencing more sexual distress might fuel avoidant‐focused cognitive processing and behaviour, thus leading to a reduction in cognitive fusion. That is, while distress and cognitive entanglement often co‐occur in the moment, elevations in distress may eventually drive individuals to disengage from or distance themselves from their thoughts. This is consistent with our observation that contemporaneous levels of sexual distress and cognitive fusion were positively correlated. However, we cannot conclude this was the case, as we have not collected data on avoidance, and particularly of the avoidance of sexual behaviour. Another possible interpretation, perhaps more parsimonious, can be drawn by considering the items of the SDS‐R, and the levels of sexual distress over the 12‐month period: firstly, sexual distress decreased over time, which may be a result of participants with higher sexual distress at baseline seeking sexual health resources, thus improving their sexual distress and reducing their entanglement with internal experiences (cognitive fusion). Help‐seeking behaviours throughout data collection should be monitored in future studies. Perhaps more importantly, the items in SDS‐R are not able to directly measure sexual distress with sexual function and sexual activity, but is instead a measure of sexual distress with sexuality more broadly, without specifying which aspects of their sexual life are the focus of distress (e.g., “Distressed about your sex life”, “Stressed about sex”, “Embarrassed about sexual problems”), thus the focus of sexual distress might not be related with endometriosis and pain. Similarly, difficulties in ER were negatively associated with sexual distress over time. To conduct an in‐depth interpretation of this result, and to test the hypothesis that a decrease in sexual distress might result from sexual avoidance (which is in line with existing literature; Fritzer et al., 2013) and avoidant‐focused ER, future studies should use a measure of sexual distress with sexual function (Mitchell et al., 2022), should monitor sexual activity and use the extended version of the DERS scale to test whether avoidant ER may lead to a reduction of sexual distress (perhaps not through a decrease in sexual problems, but rather an increase in sexual avoidance).

The contemporaneous network (Table 4) showed a strong and positive association between pain intensity and somatization, depression, and anxiety, reflecting the co‐occurrence and mutual reinforcement of these symptoms. This echoes prior research showing that psychological distress is not simply an outcome of (chronic) pain, but rather a part of an intermingled system where mental health and pain dynamically shape one another (Linton et al., 2018; Ruiz‐Aranda et al., 2010). It also seems to echo findings that suggest that women with endometriosis present more psychosomatic symptoms than control women, and those symptoms seem to correlate with increased frequency, duration, and intensity of pain (Candan et al., 2025). Additionally, the strong contemporaneous associations between sexual distress and anxiety, as well as between sexual distress and pain intensity, emphasize the immediate burden that both emotional states and physical symptoms place on an important component of the sexual health of women with endometriosis (Del Forno et al., 2024; Van Niekerk et al., 2022).

Interestingly, the contemporaneous network did not show directional effects between some of the variables, including somatization, depression, and sexual distress with ER, depression and anxiety with somatization, sexual distress and anxiety with endometriosis pain, depression with sexual distress, and ER with cognitive fusion. These patterns demonstrate possible indirect effects within the network, whereby the relationship between such variables is explained indirectly through other variables. Through our inspection of the coefficients in Table 4, we observe some combinations of potential indirect effects. For example, pain intensity has a relatively negligible bivariate and partial correlation with depression, though strong direct reciprocal effects, which suggests that additional variables may represent a common underlying cause of both. Examination of our temporal effects (Table 3) may further contextualize these potential mediation effects, wherein somatization was shown to predict pain intensity over time, endometriosis‐related pain impact predicts depression, and endometriosis‐related pain impact also significantly predicts somatization. Other potential contemporaneous mediation possibilities are outlined in Appendix C, and future investigations should empirically explore these models.

Results from the between‐subjects network analysis (Table 4) provided further nuances, showing that while some relationships were reciprocal (e.g., anxiety and depression), others were asymmetrical. For instance, while anxiety and somatization were strongly associated with sexual distress, the reverse was less pronounced. This asymmetry seems to align with previous research showing that cognitive processes associated with somatization (e.g., worry) are better predictors of sexual distress than physical symptoms of endometriosis (e.g., dyspareunia; Zarbo, Brugnera, Secomandi, et al., 2019; Zarbo, Brugnera, Compare, et al., 2019). This seems to support the biopsychosocial model of sexual health in endometriosis (e.g., Vannuccini et al., 2023), and although not refuting the bidirectionality between somatization and sexual distress (e.g., Pluchino et al., 2016), it seems to suggest that anxiety and somatization are better predictors of sexual distress than the other way around. Notably, cognitive fusion and somatization were strongly related at the between‐person level, underscoring that individuals who are more entangled with cognitive processes may experience heightened somatic symptoms, which further impacts their pain experience nefariously.

### Limitations and future directions

These results should be interpreted with caution due to the methodological limitations. Firstly, although this study follows a longitudinal design and network analyses, thus being able to explore temporality and directionality, it cannot draw causality between variables (as this is, nonetheless, an observational approach, rather than an experimental one). Future studies should employ experimental designs to better explore self‐perpetuating causal relationships between variables, and/or follow Ecological Momentary Assessment methodologies to capture short‐term dynamic fluctuations and thus more effectively identify potential causal relationships. Also, this study followed a non‐probabilistic convenience sampling, wherein participants were disproportionately highly educated (i.e., more than 70% having at least a bachelor's degree), which may not fully represent the population of women with endometriosis and therefore hinders the representativeness and generalizability of the current results. Future studies should collect data through probabilistic sampling and conduct multi‐group analyses to test whether the relationship between variables is invariant across different sociodemographic groups, to increase the likelihood of attaining a representative sample of women with endometriosis. Related to this point, it should be noted that 30% (*n* = 63) of the current sample reported having sexual problems, seemingly below other studies that found 47% of endometriosis patients with sexual dysfunction (Fagervold et al., 2009), which may be explained by social desirability, unawareness of the sexual impact of endometriosis, avoidance of sexual contact that causes problems or an under‐representation of women with endometriosis with sexual problems related to sexual function. This may have impacted the results as 70% of the sample did not report having sexual problems. Also, the SDS‐R scale does not measure the focus of sexual distress. Future studies should use a measure of sexual distress with sexual function (e.g., the Sexual Function Evaluation Questionnaire; Mitchell et al., 2022) as recent studies on the different sources of sexual distress suggest. It is also important to note that we have not collected data on the frequency of current sexual activity; thus, scores on the SDS‐R scale might have been influenced by low or no current sexual activity related or unrelated to endometriosis. Future studies should collect data not only on relationship status but also on the frequency of sexual activity, underlying motives for reduced sexual activity, and relationship satisfaction. Finally, although our study was adequately powered at the outset, attrition occurred over time, reducing the sample size at later waves (e.g., drop‐out by those who deteriorated over time and/or were more affected by the research topic). Although multiple imputation somewhat mitigated concerns about missing data within the follow‐up measurements, the usable sample sizes after the first wave were small, resulting in reduced statistical power. Future studies should be conducted in larger samples and with more frequent measurement points than three assessments over 12 months, to enhance statistical power and capture more nuanced temporal dynamics (Hecht & Zitzmann, 2021a).

Overall, this study focused on the relationship between psychological processes and symptoms, without empirically considering the broader healthcare context and related issues that may contribute to the relationships tested. For example, the psychological and sexual distress observed in this study may reflect not only individual processes but also systemic gaps in healthcare, such as diagnostic delays and insufficient multidisciplinary management of endometriosis (Pickett et al., 2023). Additionally, psychological distress related to the experience of pain may be influenced by the trivialization of these symptoms by medical professionals who may view pain as normative menstrual symptoms (Pettersson & Berterö, 2020). Future studies should empirically test models that integrate psychological factors and social factors related to systemic healthcare to provide a more holistic understanding of distress in endometriosis.

## CONCLUSIONS

Taken together, these results seem to suggest that rather than viewing pain, psychological distress, and sexual distress as isolated phenomena, they form an interconnected self‐reinforcing network of symptoms and psychological processes. Also, it adds to the growing acknowledgment that sexual distress in endometriosis is not solely determined by pain or dyspareunia (Barbara et al., 2017) but is rather impacted by complex interactions between psychological, cognitive, and emotional factors (Fritzer et al., 2013). This has important clinical implications. It suggests that transdiagnostic psychological approaches that target central nodes (e.g., cognitive fusion, emotion regulation difficulties) may result in cascading benefits across different symptomatology and components of health, such as physical health (e.g., pain), mental health (e.g., psychological distress), and sexual health (e.g., sexual distress). Overall, by recognizing these interconnected psychological and physical mechanisms, these results underscore the importance of integrated, biopsychosocial approaches within healthcare systems, which bring together psychology and sexual healthcare‐related medical specialties (e.g., gynaecology).

## AUTHOR CONTRIBUTIONS

**Sérgio A. Carvalho:** Conceptualization; investigation; writing – original draft; methodology; writing – review and editing; validation; project administration; data curation; supervision; visualization; formal analysis; software. **Brittany Patafio:** Investigation; writing – original draft; formal analysis; software; methodology; data curation. **Patrícia M. Pascoal:** Conceptualization; investigation; writing – review and editing; supervision. **Teresa Lapa:** Conceptualization; validation; writing – review and editing. **David Skvarc:** Conceptualization; methodology; writing – original draft; software; formal analysis; supervision; data curation; investigation.

## FUNDING INFORMATION

The researcher's activity was funded by national funds from FCT—Fundação para a Ciência e a Tecnologia, I.P., under the Individual Call to Scientific Employment Stimulus with ref.: 2021.01871.CEECIND (https://doi.org/10.54499/UIDP/00730/2020) (S.A.C.). The first author was partially funded by CINEICC R&D unit (https://doi.org/10.54499/UID/PRR/00730/2025). The third author’s research work is funded by the FCT through the HEI‐Lab R&D Unit (https://doi.org/10.54499/UIDB/05380/2020).

## CONFLICT OF INTEREST STATEMENT

The authors report no competing interests.

## Data Availability

The data and code that support the findings of this study are available from the corresponding author upon request.

## APPENDIX A

### Complete table of temporal effects within the network analysis

**Table:**

Predictor variable (from)	Temporal dependent variable (to)	B	SE	*p*	Random effects SD
Anxiety	Anxiety	−.688	.190	**< .001**	.219
Emotion regulation	Emotion regulation	−.596	.146	**< .001**	.232
Cognitive fusion	Cognitive fusion	−.548	.106	**< .001**	.177
Pain intensity	Pain intensity	−.401	.127	.**002**	.256
Sexual distress	Sexual distress	−.314	.123	.**011**	.226
Endometriosis pain	Endometriosis pain	−.337	.143	.**019**	.261
Somatization	Somatization	−.263	.131	.**045**	.242
Sexual distress	Cognitive fusion	−.318	.096	.**001**	.184
Endometriosis pain	Cognitive fusion	.320	.103	.**002**	.170
Endometriosis pain	Depression	.321	.139	.**021**	.262
Emotion regulation	Sexual distress	−.308	.135	.**023**	.219
Somatization	Pain intensity	−.283	.134	.**035**	.259
Pain intensity	Sexual distress	−.253	.124	.**040**	.220
Endometriosis pain	Somatization	.269	.135	.**046**	.243
Cognitive fusion	Emotion regulation	.255	.147	.082	.264
Anxiety	Sexual distress	−.379	.220	.085	.212
Pain intensity	Anxiety	−.171	.109	.117	.215
Depression	Sexual distress	.358	.231	.120	.209
Endometriosis pain	Anxiety	.179	.118	.128	.216
Depression	Depression	−.351	.232	.131	.233
Endometriosis pain	Emotion regulation	.207	.144	.149	.251
Sexual distress	Anxiety	−.156	.110	.154	.215
Anxiety	Pain intensity	.291	.223	.192	.251
Depression	Somatization	−.273	.225	.227	.282
Somatization	Cognitive fusion	−.120	.099	.228	.184
Emotion regulation	Anxiety	−.138	.120	.251	.215
Anxiety	Depression	−.246	.225	.273	.251
Somatization	Endometriosis pain	−.142	.140	.308	.258
Depression	Anxiety	.200	.197	.309	.219
Somatization	Sexual distress	−.126	.126	.316	.235
Endometriosis pain	Sexual distress	.128	.131	.328	.191
Pain intensity	Endometriosis pain	−.125	.132	.346	.261
Pain intensity	Depression	−.119	.129	.356	.231
Emotion regulation	Pain intensity	−.120	.141	.393	.243
Sexual distress	Emotion regulation	.114	.134	.395	.277
Sexual distress	Endometriosis pain	−.095	.134	.478	.261
Pain intensity	Emotion regulation	−.093	.132	.481	.309
Emotion regulation	Somatization	−.096	.138	.485	.242
Depression	Pain intensity	−.158	.231	.495	.253
Depression	Emotion regulation	−.152	.237	.521	.292
Endometriosis pain	Pain intensity	.083	.138	.545	.262
Sexual distress	Depression	−.070	.130	.593	.256
Cognitive fusion	Depression	−.076	.143	.593	.261
Somatization	Anxiety	.053	.115	.646	.210
Sexual distress	Pain intensity	−.056	.129	.662	.243
Depression	Cognitive fusion	−.073	.175	.676	.185
Cognitive fusion	Pain intensity	.058	.141	.681	.243
Cognitive fusion	Sexual distress	.056	.137	.684	.193
Sexual distress	Somatization	−.049	.126	.696	.244
Somatization	Emotion regulation	.054	.140	.701	.258
Anxiety	Cognitive fusion	−.064	.168	.706	.170
Anxiety	Somatization	−.082	.218	.707	.242
Cognitive fusion	Anxiety	−.044	.121	.716	.215
Emotion regulation	Endometriosis pain	.050	.146	.732	.272
Pain intensity	Cognitive fusion	−.032	.096	.742	.183
Pain intensity	Somatization	−.040	.125	.749	.242
Somatization	Depression	.043	.135	.751	.268
Anxiety	Endometriosis pain	.062	.230	.789	.278
Cognitive fusion	Somatization	−.034	.138	.807	.243
Cognitive fusion	Endometriosis pain	−.034	.146	.817	.270
Emotion regulation	Cognitive fusion	.019	.105	.854	.194
Anxiety	Emotion regulation	−.032	.229	.888	.265
Emotion regulation	Depression	.009	.142	.950	.262
Depression	Endometriosis pain	.002	.237	.994	.270

*Note*: B represents the standardized regression coefficient *from* a variable at earlier measurements *to* future, representing the average effect for that variable across the second and third measurement. Significant values are indicated in bold.

## APPENDIX C

### Possible mediation effects present in the data

**TABLE C1:** Possible temporal mediation effects.

Independent variable	Mediator variable	Dependent variable
Pain intensity	Sex distress	Cognitive fusion
Emotion regulation		
Somatization	Pain intensity	Sex distress
Endometriosis pain	Somatization	Pain intensity

**TABLE C2:** Possible contemporanous mediation effects.

Independent variable	Mediator variable	Dependent variable
Anxiety	Pain intensity	Somatization
	Sex distress	
Anxiety	Pain intensity	Depression
	Cognitive fusion	
Sex distress	Pain intensity	Depression
Emotion regulation	Pain intensity	Somatization
Cognitive fusion	Anxiety	Sex distress
	Somatization	
Endometriosis pain	Pain intensity	Anxiety
	Cognitive fusion	

**TABLE C3:** Possible between‐subjects mediation effects.

Independent variable	Mediator variable	Dependent variable
Depression	Sex distress	Somatization
Somatization	Endometriosis pain	Depression
Anxiety	Cognitive fusion	Somatization
	Sex distress	
Anxiety	Pain intensity	Emotion regulation
Depression	Sex distress	Cognitive fusion
Pain intensity	Anxiety	Cognitive fusion
Endometriosis pain	Depression	Pain intensity
Pain intensity	Anxiety	Endometriosis pain
